# A rapid multi-disciplinary biodiversity assessment of the Kamdebooberge (Sneeuberg, Eastern Cape, South Africa): implications for conservation

**DOI:** 10.1186/2193-1801-1-56

**Published:** 2012-12-06

**Authors:** Vincent R Clark, Sandun J Perera, Michael Stiller, Charles H Stirton, Peter H Weston, Pavel Stoev, Gareth Coombs, Dale B Morris, Dayani Ratnayake-Perera, Nigel P Barker, Gillian K McGregor

**Affiliations:** 1 Department of Botany, Rhodes University, Grahamstown, 6140 South Africa; 2 School of Environmental Sciences, University of KwaZulu-Natal, Westville Campus, Durban, 4000 South Africa; 3 Biosystematics Division, Agricultural Research Council, Plant Protection Research Institute, Private Bag X134, Pretoria, Queenswood, 0121 South Africa; 4 Department of Botany, University of Cape Town, Rondebosch, 7700 South Africa; 5 National Herbarium of New South Wales, Sydney, 2000 Australia; 6 National Museum of Natural History, Sofia and Pensoft Publishers, Sofia, Bulgaria; 7 Department of Zoology and Entomology, Rhodes University, Grahamstown, 6140 South Africa; 8 Department of Geography, Rhodes University, Grahamstown, 6140 South Africa

**Keywords:** Endemics, Great escarpment, Kamdebooberge, Plants, Invertebrates, Sneeuberg centre of floristic endemism, Vertebrates

## Abstract

Botanical work since 2008 on the Sleeping Giant section of the Kamdebooberge (Sneeuberg mountain complex, Eastern Cape, South Africa) has indicated that these mountains may be of significant conservation value. Accordingly, a precursory, rapid multi-disciplinary biodiversity assessment was undertaken in January 2011, focusing on plants, tetrapod vertebrates and leafhoppers. The botanical results confirm the Kamdebooberge as being of high botanical conservation value, hosting three strict endemics, healthy populations of five other Sneeuberg endemics, and fynbos communities comprising species not found elsewhere in the Sneeuberg. The Kamdebooberge are important for herpetofauna (excluding serpentoids) and mammals, hosting several range-restricted and regional endemics. The expedition uncovered three new leafhopper species, together with several species previously only known from the Cape Floristic Region. Further detailed faunal work may provide further interesting results from these mountains, which show a high conservation value unique to the southern Escarpment.

**Electronic supplementary material:**

The online version of this article (doi:10.1186/2193-1801-1-56) contains supplementary material, which is available to authorized users.

## Introduction

**Figure 1:**
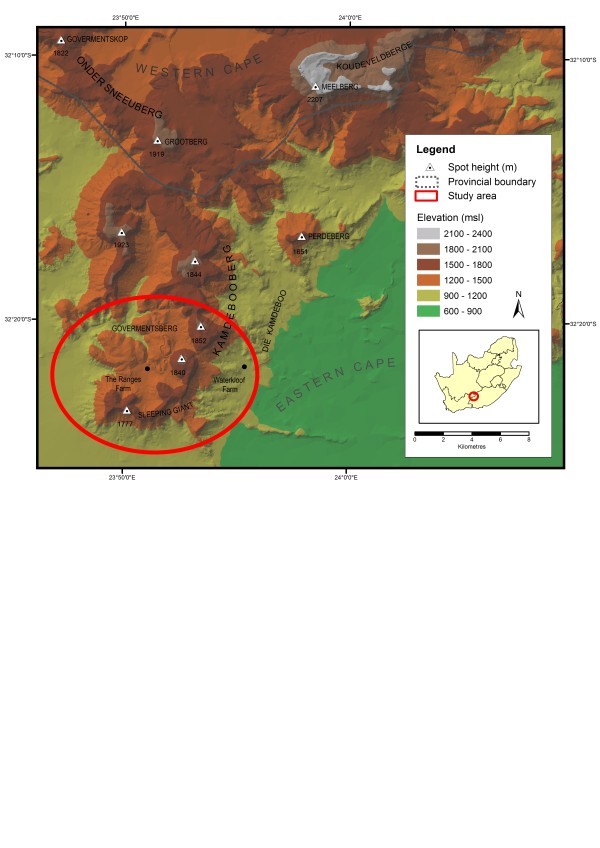
**The location of the Kamdebooberge (Sleeping Giant section), Sneeuberg mountain complex, indicating the field survey area.**

The Sleeping Giant section of the Kamdebooberge forms the south-western end of the arc-shaped Sneeuberg mountain complex, in the Eastern Cape Province of South Africa (Figure 1). The Sneeuberg forms part of the overall poorly explored southern African Great Escarpment, and was recently recognised as a new centre of floristic endemism (Clark et al. 2009,2011; Figure 1), and as a distinct zoogeographical unit within the Greater Maputaland–Pondoland–Albany region of vertebrate endemism (Perera et al. 2011). The Kamdebooberge themselves have become increasingly interesting following the discovery in 2008 of two new, very localised plant taxa, two of which belong to genera previously unknown from these drier southern Great Escarpment mountains (e.g. Williams 1982; Rebelo 2001). Apart from two narrow-endemic butterfly species (*Cassionympha camdeboo* and *Thestor camdeboo*; Woodhall 2005) and a few bird records, not much is known about the fauna of the Kamdebooberge.

Due to absence of any previous faunal research on the Kamdebooberge, and the potential for further important botanical findings, a multi-disciplinary team of ten biodiversity scientists visited the Kamdebooberge from 22–25 January 2011. The purpose was a precursory, rapid biodiversity survey of the southern section of the Kamdebooberge, focusing on the disciplinary skills of each scientist, and to obtain an indication of the conservation value and natural heritage of these mountains. Results indicated high levels of endemism in animals, with some links to adjoining biomes. For the flora, more records of endemics were established in a poorly explored region. This multidisciplinary approach serves as an example for future research in the poorly explored Great Escarpment.

### The study area

A detailed overview of the Sneeuberg Centre of Floristic Endemism and the Great Escarpment is provided by Clark et al. (2009,2011 respectively) and provides the broader context for this study.

**Figure 2:**
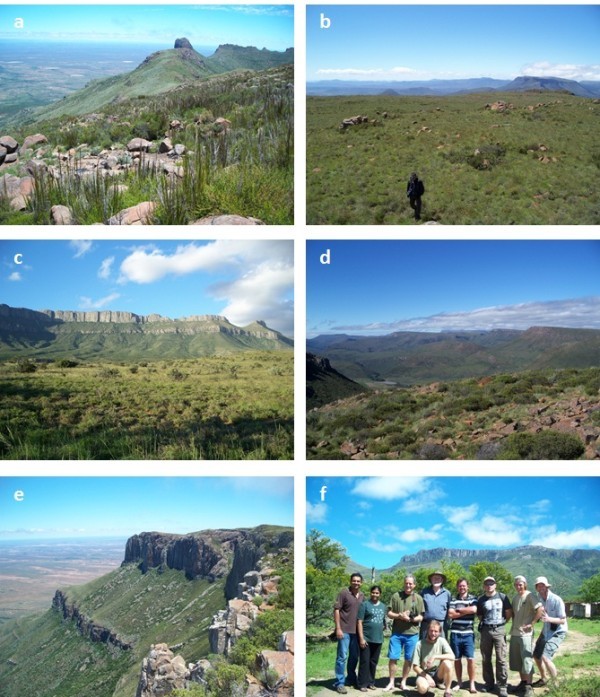
**The Kamdebooberge: a fynbos communities; b summit grassland; c & d the southern scarp; e panoramic view looking north-west over ‘The Ranges’ towards the Meelberg (far distance); f the Team, from left to right: Sandun & Dayani Perera, Peter Weston, Charlie Stirton, Mike Stiller (front), Gareth Coombs, Pavel Stoev, Dale Morris, Ralph Clark.** Goewermentsberg in the background. Photo credits: Ralph Clark.

The Kamdebooberge itself covers some 170 km^2^ and comprises a dissected plateau-spur off the higher (2,100–2,300 m) Meelberg–Koudveldberge–Toorberg plateau to the north (Figures 1, 2). It is characterised by isolated, gently sloping plateaus (altitude 1,600–1,900 m) connected by cols and often bounded by vertical cliffs. The geology is comprised of shales and sandstones of the Beaufort Group, heavily intruded by dolerites. The more resistant dolerite sills and dykes have given the mountain range a characteristic shape, and when viewed from the east looks like a ‘Sleeping Giant’, as named accordingly (Chief Director of Surveys and Mapping 1987). The Farms visited were Plaas 96, 97, 98 and 99, and parts of The Ranges 69 and Oaklands 104 (the area between 32^o^20^′^S to 32^o^24^′^S, and 23^o^50^′^E to 23^o^53^′^E).

Mean annual rainfall at the base of the Kamdebooberge (Farm Waterkloof) is ca. 400 mm, and on the south-east-facing slopes of the mountain is predicted to be at least 700–800 mm due to orographic effect as evidenced by the incipient forest and afromontane grassland and fynbos. Rainfall on the mountain is augmented by regular mist, and snowfalls occur most winters. Exceptional rains had fallen in the Kamdeboo Karoo since the middle of December 2010, breaking a severe two-year drought, and the weather during the expedition was a combination of heavy morning mist, heavy evening showers, and sunshine.

From four previous botanical trips to the area by VRC, several vegetation types can be defined as occurring on the Kamdebooberge, providing a variety of habitats for fauna and flora:

-   Drier thicket/closed woodland occupies the north-facing slopes and lower southern/south-eastern slopes. Typical species are *Acacia karroo* Hayne, *Aloe ferox* Mill., *Buddleja glomerata* H.L.Wendl., *Carissa haematocarpa* (Eckl.) A.DC., *Diospyros lycioides* Desf., *Dodonaea viscosa* var. *angustifolia* (L.f.) Benth, *Ehretia rigida* (Thunb.) Druce subsp. *rigida*, *Euclea crispa* (Thunb.) Gürke subsp. *crispa*, *Gymnosporia buxifolia* (L.) Szyszyl., *Olea europaea* subsp. *africana* (Mill.) P.S.Green, *Searsia lancea* (L.f.) F.A.Barkley and *S. pallens* (Eckl. & Zeyh.) Moffett*.*
-  Mesic thicket and closed woodland occur on the moister mid-altitude slopes. Typical species are *Celtis africana* Burm.f., *Cussonia spicata* Thunb., *Kiggelaria africana* L., *Leucosidea sericea* Eckl. & Zeyh., *Searsia longispina* (Eckl. & Zeyh.) Moffett and *S. pyroides* (Burch.) Moffett.
-  Very localised ‘pre’-afromontane forest occurs on the SE-facing slope of Goewermentsberg. Typical species are *Buddleja salviifolia* (L.) Lam., *Diospyros scabrida* var. *cordata* (E.Mey. ex A.DC.) De Winter, *Grewia occidentalis* L. var. *occidentalis*, *Heteromorpha arborescens* (Spreng.) Cham. & Schltdl. var. *arborescens* (interior form), *Kiggelaria africana* L., *Maytenus acuminata* (L.f.) Loes. var. *acuminata*, *M. undata* (Thunb.) Blakelock, *Olinia emarginata* Burtt Davy, *Pittosporum viridiflorum* Sims, *Searsia dentata* (Thunb.) F.A.Barkley and *S. rehmanniana* var. *glabrata* (Sond.) Moffett.
-  Closed shrubland dominates the drier mid-slopes, and is characterised by species such as *Elytropappus rhinocerotis* L.f., *Euryops annae* E.Phillips and *Otholobium macradenium* (Harv.) C.H.Stirt.
-  A very species-rich afromontane grassland-shrubland mosaic occurs on the moister mid- to upper altitude slopes and summit plateau, and is mixed with numerous fynbos elements (see below). The dominant grasses are *Themeda triandra* Forssk. and *Merxmuellera disticha* (Nees) Conert. Several Sneeuberg endemics are common to abundant, such as *Euryops dentatus* B.Nord., *Gazania caespitosa* Bolus and *Psoralea margaretiflora* C.H. Stirt. & V.R.Clark.
-  Pure stands of fynbos occur in fire-refugia, and are typically dominated by species such as *Acmadenia* sp. nov., *Agathosma* sp., *A. venusta* (Eckl. & Zeyh.) Pillans, *Cliffortia montana* Weim., *Clutia alaternoides* L., *Erica leucopelta* Tausch, *E. passerinoides* (Bolus) E.G.H.Oliv., *Ficinia nigrescens* (Schrad.) J.Raynal, *Ischyrolepis* sp. aff. *constipata* H.P.Linder, *Phylica paniculata* Willd., *Rhodocoma capensis* Steud., *R. fruticosa* (Thunb.) H.P.Linder, *Tetraria cuspidata* (Rottb.) C.B.Clarke and *T. fourcadei* Turrill & Schönland. These fynbos elements are particularly interesting in that some of these species are disjunctions from the Cape Floristic Region (hereafter CFR).
-  Localised habitats (micro-habitats) occur, the mostly typical being summit wetlands and cliff-lines. Wetlands are characterised by *Kniphofia caulescens* Baker and *Merxmuellera macowanii* (Stapf) Conert), and cliff-lines by a variety of lithophytic ferns such as *Asplenium adiantum-nigrum* L. var. *adiantum-nigrum*, *A. trichomanes* subsp. *quadrivalens* D.E.Mey., *Cystopteris fragilis* (L.) Bernh., and succulents such as *Crassula perforata* Thunb., *Haworthia marumiana* var. *batesiana* (Uitewaal) M.B.Bayer, *Othonna patula* Schltr. and *Senecio articulatus* (L.f.) Sch.Bip.

## Methods

### Plants

As the Kamdebooberge falls into one Quarter Degree Grid Square (3223BD), the intention was not to collect every species encountered, but to rather complement previous comprehensive work done on the adjacent Goewermentsberg since 2008. Thus species not previously collected in the Kamdebooberge were prioritised over other species. The vegetation was in excellent condition following the exceptional recent rainfalls, a previous burn on some of the plateau, and the absence of livestock grazing.

Plants on the mostly uniform summit plateau were sampled by traversing the plateau on foot, as an approximation of the line transect method (Buckland et al. 2007). Micro-habitats (rock outcrops, seeps, cliff-lines, dolerite boulder-fields) were more intensively sampled on an *ad hoc* basis by careful visual examination. Plants were pressed as per standard practice and later identified in the Selmar Schonland Herbarium (GRA), Albany Museum, Grahamstown, with some specimens sent to taxonomists for expert identification (see Acknowledgements). Apart from these latter specimens, all specimens are now lodged in GRA, with duplicates having been sent to the Bolus Herbarium (University of Cape Town, BOL), the National Herbarium of New South Wales (Australia, NSW), the Royal Botanical Gardens, Kew (K), Missouri Botanical Gardens (MO), the University of Stellenbosch Herbarium (STEU), and the Swedish Museum of Natural History (S).

### General fauna

A preliminary rapid survey on the Kamdebooberge fauna was conducted while traversing the summit plateau on foot. The rapid assessment methods included opportunistic observations on amphibians, birds and mammals; active searches (Garden et al. 2007) for reptiles; and random collection and photography of common invertebrates. Particular emphasis was given to looking for the bird *Chaetops aurantius* (Drakensberg Rockjumper) for the purposes of obtaining DNA samples of this Eastern Cape Escarpment and Drakensberg/Maluti endemic (Hockey et al. 2005).

Rodent trapping was attempted using 30 Sherman traps, but logistical difficulties and the preliminary nature of the expedition frustrated trapping on the summit plateau itself. Instead, traps were laid in three transects of ten traps each (ten metres apart) at 1,320 m on the mid-altitude plateau some 500 m east of the farmhouse ‘The Ranges’, in disturbed, closed shrubland and seasonal marshland, for one night (∑*c.* 300 trapping hours), using peanut butter with chopped vegetables as the bait.

### Leafhoppers and planthoppers

Leafhoppers (Hemiptera: Cicadomorpha: Cicadellidae) and planthoppers (Hemiptera: Fulgoromorpha) were the only invertebrate groups sampled systematically. The method used to collect leafhoppers was the traditional sweep net. Mechanised methods such as vacuum sampling could not be used due to the inaccessibility of the terrain. Two tree species were sampled by fogging with a pyrethroid pesticide in the foothills. Identification of leafhoppers was undertaken by MS at the National Collection of Insects, Biosystematics Division of the Agricultural Research Council, using dissections, published descriptions, and comparing with available described species and undescribed specimens housed in this institution.

## Results and discussion

### Plants

Ninety-seven plant specimens were collected, representing 92 species (Additional file 1: Appendix 1). Five of the species collected – *Albuca tortuosa* Baker, *Cyperus tabularis* Schrad., *Disa porrecta* Sw., *Microchloa kunthii* Desv. and *Syringodea concolor* (Baker) M.P.de Vos – are additions to Clark et al.’s (2009) flora of the Sneeuberg. An updated version of the Sneeuberg flora is available on the Great Escarpment Biodiversity Programme website: http://thegreatescarpment.110mb.com.

A new population of the Kamdebooberge endemic Rutaceae species (*Acmadenia* sp. nov.) was discovered on the south-east-facing, upper scarp slope on the Farm Ossehoek 99. Fruiting material was collected for the first time and has been sent to BOL for description and publication of the species.

*Psoralea margaretiflora* C.H.Stirt. & V.R.Clark (Stirton et al. 2011) – first collected in 2005 in the eastern Sneeuberg – was verified in the field by CHS as being a new species, and a manuscript was prepared on site from the Kamdebooberge specimens. The plant is locally abundant in the eastern and western sections of the Sneeuberg. *Erica passerinoides*, originally only known from the Toorberg (20 km to the north; Clark et al. 2009), was collected again on the Kamdebooberge (a second, large population was discovered on Goewermentsberg in 2010). The Kamdebooberge may thus actually represent the core distribution of this poorly-known western Sneeuberg endemic.

New records on species until recently considered exclusively CFR species were obtained. New populations of *Rhodocoma capensis* and *Tetraria fourcadei* were encountered on the Farm Ossehoek 99, the former being the dominant species in Kamdooberge mountain fynbos. Both species are known from Goewermentsberg, where they are abundant and were the first records of these species outside of the CFR.

A massive population (i.e. several hundred thousand individuals) of the shrub *Otholobium macradenium* was encountered on the north-east facing slopes below the summit plateau on Farms Plaas 96 and The Ranges 69 (1,300–1,700 m). This was the first time this species has been collected in flower in the Sneeuberg since the discovery of the initial Sneeuberg population in December 2005. The species is rare in the CFR (where it has only been sporadically collected) but is abundant in certain areas of the Sneeuberg, notably the Kamdebooberge and from the Nardousberg eastwards to Buffelshoek-se-Pas behind Pearston. The majority of the total population thus seems to be in the Sneeuberg.

*Cliffortia montana* was recorded as abundant and forming almost pure stands on the Farm Ossehoek 98. This species has a patchy distribution, occurring on the Swartberg (in the CFR), the western Sneeuberg (Kamdebooberge to the Toorberg) and then on the Nardousberg massif.

### General Fauna

**Figure 3:**
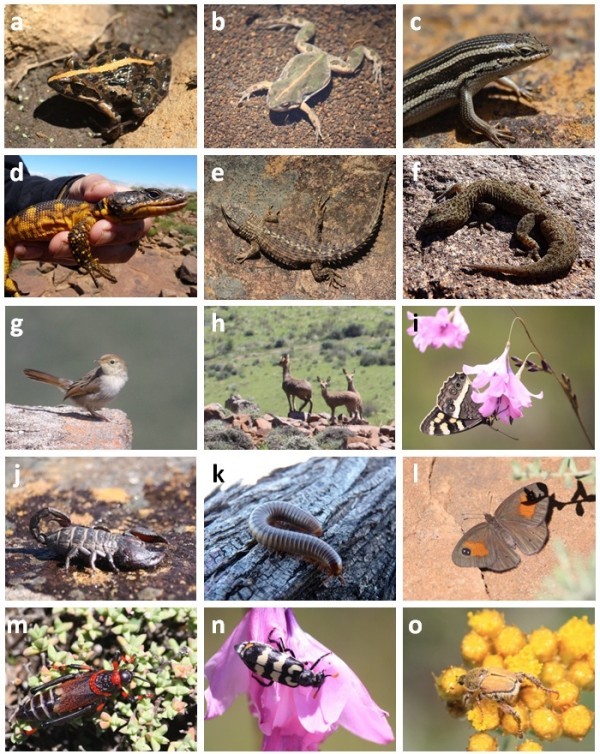
**Selection of photographs of fauna encountered during the survey: a*Strongylopus grayii*(Clicking Stream Frog), b*Cacosternum boettgeri*(Common Caco), c*Trachylepis homalocephala*(Red-sided Skink), d*Pseudocordylus microlepidotus*(Cape Crag Lizard), e*Cordylus cordylus*(Cape Girdled Lizard), f*Afroedura karroica*(Karoo Flat Gecko), g*Cisticola lais*(Wailing Cisticola), h*Oreotragus oreotragus*(Klipspringer), i*Aeropetets tulbaghia*(Mountain Pride), j*Hadogenes*sp., k*Orthoporoides*sp., l*Neita durbani*(D’Urban’s Brown), m*Dictyophorus spumans*, n*Decapotoma lunata*, o*Heterochelus*sp.** Photo credits: a–c, e, g–j, l–o Sandun Perera; d, f & k Pavel Stoev.

A total of 24 tetrapod vertebrate species were recorded (Additional file 2: Appendix 2, Figure 3), in addition to several other invertebrates recorded incidentally (except the leafhoppers, addressed separately).

The vertebrates included three species of amphibians recorded on the summit; *Amietophrynus rangeri* (Raucous Toad), *Cacosternum boettgeri* (Dainty Frog) and *Strongylopus grayii* (Clicking Stream Frog). Both *Strongylopus grayii* and *Cacosternum boettgeri* were recorded from a streamlet on the summit plateau, also indicating the watershed value of the Kamdebooberge. Though not recorded from the summit (hence not included in the list) *Tomopterna tandyi* (Tandy’s Sand Frog) was found from the foothills of the Kamdebooberge, on Farm Oaklands 104.

Five species of reptiles were recorded from the summit, among which *Cordylus cordylus* (Cape Girdled Lizard), *Trachylepis homalocephala* (Red-sided Skink) and *Afroedura karroica* (Karoo Flat Gecko) were the most common. *Pachydactylus maculatus* (Spotted/Thick-toed Gecko) was observed only in the lower reaches and excluded in the list. Serpentoid reptiles were conspicuous in their absence from the summit plateau.

Avifauna on the mountain was sparse, with only twelve species recorded during the survey. Apart from raptors and two southern African endemics, these were mostly inconspicuous birds. Three of the raptors – *Buteo vulpinus* (Steppe Buzzard), *Falco amurensis* (Amur Falcon) and *Milvus migrans* (Yellow-billed Kite) – are non-breeding migrants and not confined to mountain regions (Hockey et al. 2005). No evidence (visual or audio) of *Chaetops aurantius* (Drakensberg Rockjumper) was noted, although the habitat is favourable. As the bird was recorded visually on the higher Koudeveldberge massif (20 km to north) in December 2011 by VRC, there is a chance that it may occur sporadically on these lower Kamdebooberg mountains. The greatest number of bird species were noted from the surrounding Karoo plains (not included in the Additional file 2: Appendix 2), most spectacularly an influx of *Neotis ludwigii* (Lüdwig’s Bustard) feeding on the emergent insects during and after the heavy rains.

Four mammals species were recorded, namely *Hystrix africaeaustralis* (Cape Porcupine), *Oreotragus oreotragus* (Klipspringer), *Pelea capreolus* (Grey Rhebok) and *Procavia capensis* (Rock Hyrax/ Dassie). Rodent trapping was unsuccessful, possibly due to inclement weather, the trapping period being too short, and the absence of pre-baiting.

An interesting array of invertebrates was noted on the summit plateau. A rich butterfly fauna is evident, with *Aeropetes tulbaghia* (Mountain Pride) being one of the most conspicuous species seen. Several specimens were collected for Garreth Keevey’s systematics research on this CFR–eastern Great Escarpment endemic (Woodhall 2005). Other interesting species recorded were *Tarucus thespis* (Fynbos Blue; found in CFR, Sneeuberg and Great Winterberg–Amatolas) and *Neita durbani* (D’Urban’s Brown; an Eastern Cape endemic; Woodhall 2005). Among the other invertebrates recorded were four myriapods two species of the genus *Orthoporoides* (Spirostreptida) and one of Polydesmida (Diplopoda), and *Rhysida afra* (Chilopoda: Scolopendromorpha); a *Hadogenes* species (scorpion); grasshoppers such as *Dictyophorus spumans* and *Scintharista* cf. *saucia*; the blister beetle *Decapotoma lunata*, the dung beetle *Macroderes bias*, and a monkey beetle of the genus *Heterochelus*.

### Leafhoppers

Recent work on grass-feeding endemic leafhoppers in the Fynbos and Grassland Biomes in South Africa (Durr 1988; Stiller [Bibr CR20_45][Bibr CR21_45][Bibr CR22_45][Bibr CR23_45][Bibr CR24_45][Bibr CR25_45]) has produced three new genera and 61 new species. Many forb, shrub and tree-associated leafhoppers are still awaiting description. The total number of leafhoppers in southern Africa is estimated conservatively at 600 species, up considerably from the estimated 350 of Scholtz and Holm (1985).

**Figure 4:**
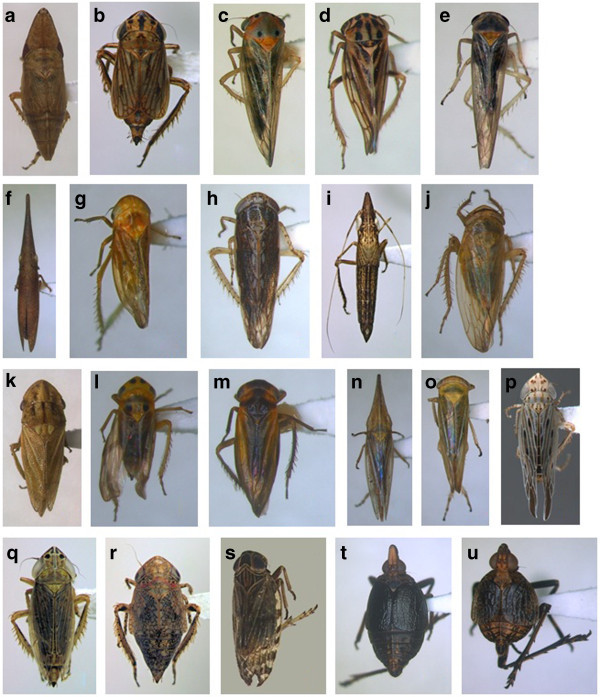
**Leafhoppers and planthoppers collected on the Kamdebooberge (average length of specimens 5 mm): a*Afralycisca umbrina*, b n.gen & sp.1, Athysanini, c*Austroagallia*sp1, d*Austroagallia*sp2, e*Balclutha*sp., f*Cephalelus attenuatus*, g*Chlorita cylindrica*, h*Circulifer struthiola*, i*Drakensbergena gigascutica*, j*Exitianus taeniaticeps*, k*Hangklippia signata*, l*Iseza*sp., m*Molopopterus*sp., n*Paradorydium*sp., o*Tetartostylus*sp., p*Teinopterus mikrophallus*, q*Tetramelasma litopyx*, r*Tzitzikamaia*sp., s Cixiidae, t*Menenches atropos*, u*M. decuma.***

Thirty-nine species were recorded on the Kamdebooberge (Additional file 3: Appendix 3, Figure 4), with at least three well documented Grassland Biome endemics. Records of widespread species include pests or potential pests that could transmit plant viruses. These widespread species include *Accacidia improvisa* (first described from Egypt and Sudan on *Acacia*), *Austroagallia*, *Chlorita*, *Circulifer*, *Coloborrhis*, *Empoasca*, *Exitianus*, *Naudeus*, *Paradorydium*, *Recilia*, *Tetartostylus* and *Vilargus*. The species of *Chlorita* are difficult to distinguish, with *C. exilis*, described by Theron (1977,1986,1988) from specimens only found in the CFR on *Elytropappus rhinocerotis* and *Seriphium plumosum* L. (both Asteraceae). *Chlorita cylindrica* was redescribed (Theron 1988) from many records in the Western Cape, Eastern Cape and Free State, with *Chrysocoma ciliata* L. (Asteraceae) as host from some of these records. *Iseza* is characterised by distinct marking on the head, but species are difficult to distinguish. *Naudeus bivittatus* is known to feed on the grass *Imperata cylindrica* (L.) Raeuschel in South Africa (this grass was not recorded on this expedition but is known from the Sneeuberg region; Clark et al. 2009). *Tzitzikamaia* sp. cannot be identified further as only nymphs and females were collected; three species of this genus are known from South Africa. *Molopopterus damus* was described from Swellendam, Western Cape, and the Kamdebooberge specimen may represent a disjunction onto the southern Great Escarpment from the CFR. *Molopopterus obliquus* Theron was described in 1978 from a long series of specimens from Jonkershoek, near Stellenbosch (Western Cape Province) on *Otholobium obliquum* (E. Mey.) C.H.Stirt. The survey specimens were swept from *Otholobium macradenium* and *Passerina montana*, and may also represent a disjunction from the CFR onto the southern Great Escarpment. Uncertainty within *Molopopterus* species can be seen in Dworakowska (1973,1974,1981) with 22 new species from Central Africa, on grasses. Einyu and Ahmed (1983) described one species and Ahmed (1979) two species from Uganda. Theron (1978) described 27 species from South Africa, on shrubs, based on records from the CFR.

Rare or endemic species feeding on Monocotyledons includes the following: *Cephalelus attenuatus* is one of 18 species from the CFR, and this genus is always associated with one or more species of Restionaceae (of which at least four species occur in the Kamdebooberge). Records of *C. attenuatus* suggest a wide distribution in the western and southern parts of South Africa. *Drakensbergena gigascutica*, *Teinopterus mikrophallus* and *Tetramelasma litopyx* are endemic to Karoo Escarpment Grassland (Vegetation Unit GH1 of Mucina and Rutherford 2006), which occurs patchily along the southern Great Escarpment from the eastern Nuweveldberge to the Great Winterberg–Amatolas and Stormberg (Mucina and Rutherford 2006).

Dicotyledon-associations include the following: *Afralycisca umbrina* is known from few specimens and localities in South Africa. It is probably associated with forbs and appears to be more common on mountain tops in the Grassland Biome. *Hangklippia signata* is rare, based on the dearth of specimens. The new species of *Modderena* (sp. nov. near *M. albicosta* Theron in Additional file 3: Appendix 3) belongs to a genus that feed on shrubs and forbs that are relatively common in South Africa, but this species is not known from outside the Kamdebooberge. The three new genera and species in Athysanini show some similarities with species described from the CFR as well as a number of undescribed species from the Drakensberg, Soutpansberg, and mountains further north (e.g. some examined specimens from Kilimanjaro). Species 1 (#1 in Additional file 3: Appendix 3) bears some resemblance to a number of species described from the CFR. This leafhopper has well developed hind-wings, suggesting an ability to migrate, and close similarity is found in undescribed species of specimens from Storms River, Port Elizabeth, Kareedouw, Graaff-Reinet, Cradock and Fouriesburg, suggesting a wider range of this species than the Kamdebooberge. Species 2 has a reduced hind-wing that is about 1/3 as long as the forewing, suggesting a poor ability to fly, and no specimens with similar features have been found in any South African insect collection. Species 3 is known only from Farm Oaklands 104, at the base of the Kamdebooberge in mixed woodland/ thicket.

In planthoppers, the dictyopharids and nogodinids probably feed on forbs and are difficult to collect by sweeping, and are probably South African endemics. Poor flying ability or lack thereof appears to be a feature of these endemics. Some planthoppers collected include short-winged species such as *Turneriola* sp., *Telmessodes proconsul* (known from the Eastern Cape), and *Menenches decuma* and *M. morta* (both described from the Drakensberg in KwaZulu-Natal).

### Remarks on conservation value

The results confirm the botanical importance of the Kamdebooberge, with its two strict endemics (*Acmadenia* sp. nov., *Faurea* sp. nov.), healthy populations of five other Sneeuberg endemics (*Erica passerinoides*, *Euryops dentatus*, *Gazania caespitosa*, *Haworthia marumiana* var. *batesiana*, *Psoralea margaretiflora*), and the unique composition of the fynbos communities found on the upper SE-facing slopes. Further botanical exploration in these mountains may well yield more undescribed species, additional populations of local endemics, and additions to the Sneeuberg flora.

Although only the list for herpetofauna (except for serpentoid reptiles) could be considered fairly complete, the results indicate that the Kamdebooberge has a rich and varied faunal diversity. Among amphibians, *Amietophrynus rangeri* (Raucous Toad) and *Strongylopus grayii* (Clicking Stream Frog) are southern African endemics (Minter et al. 2004). Out of the five species of reptiles recorded, two are of high conservation importance. The *Afroedura karroica* (Karoo Flat Gecko) is endemic to the Sneeuberg range and its immediate surroundings, while *Cordylus cordylus* (Cape Girdled Lizard) is an Eastern Cape and Western Cape endemic. *Pseudocordylus microlepidotus* (Cape Crag Lizard) and *Trachylepis homalocephala* (Red-sided Skink are southern African endemics (Branch 1998). Among the birds recorded, *Macronyx capensis* (Orange-throated Longclaw) and *Serinus canicollis* (Cape Canary) are southern African endemics (Hockey *et al.*^2005^). The importance of the Kamdebooberge for raptors-especially as a possible nesting site is evident, particularly for rugged terrain species such as *Aquila verreauxii* (Verreaux’s/Black Eagle), and from previous sightings of *Aquila pennata* (Booted Eagle) and *Polemaetus bellicosus* (Martial Eagle). From a conservation perspective the most important among recorded mammals is the South African endemic *Pelea capreolus* (Grey Rhebok), given its patchy montane distribution and its population stronghold in the Sneeuberg (Skinner and Chimimba 2005).

The total number of leafhopper species recorded is comparatively low, probably as a limited number of plants were sampled, the weather was not optimal for collecting, and the expedition took place early in the season. Furthermore, the apparent absence of fire might have an influence on leafhopper diversity, in contrast to the regularity of fire in the Grassland and Fynbos Biomes. The majority of species however are widespread throughout Africa or southern Africa, feeding on a wide range of plants, with some related to species that are sporadic or common pests in agriculture. The three undescribed species have so far only been recorded from the Kamdebooberge, and may have some affiliations with species from the CFR.

The faunal studies are still largely incomplete and demand intensive observations in future – including extensive trapping, nocturnal sampling and recording indirect evidence. Although preliminary, these results have however already sparked interest among zoologists (e.g. Myriapodes), who may pursue their own field surveys in the Kamdebooberge.

## Supplementary online data available

**Web-album:**https://picasaweb.google.com/116442385816096754015/BiodiversityScientistsExploreThePoorlyKnownKamdeboobergeInTheGreatKaroo

**Web-article:** The Great Escarpment Biodiversity Research Programme http://thegreatescarpment.110mb.com/.

## Electronic supplementary material

Additional file 1: **Appendix 1.** Plant taxa collected in the Kamdebooberge (22–25 January 2011). (DOC 104 KB)

Additional file 2: **Appendix 2.** Tetrapod vertebrates recorded from the Kamdebooberge (22–25 January 2011). (DOC 44 KB)

Additional file 3: **Appendix 3.** Leafhoppers (Cicadellidae), planthoppers (Dictyopharidae, Nogodinidae, Tropiduchidae) and treehoppers (Membracidae) collected on the Kambebooberge (22–25 January 2011). (DOC 54 KB)
