# Supplementary material for: A rapid multi-disciplinary biodiversity assessment of the Kamdebooberge (Sneeuberg, Eastern Cape, South Africa): implications for conservation
Source: Springerplus. 2012 Dec 6;1(1):56. doi: 10.1186/2193-1801-1-56 (PMC3540356; doi:10.1186/2193-1801-1-56)
Supplement: Supplementary file 1 — Additional file 1: Appendix 1. Plant taxa collected in the Kamdebooberge (22–25 January 2011). (DOC 104 KB) [file 40064_2012_45_MOESM1_ESM.doc]

Appendix 1: Plant taxa collected in the Kamdebooberge (22–25 January 2011).

| **Family** | **Taxon** | **Clark VR, Stirton CH**  **& Weston P No.** |
| --- | --- | --- |
| Aizoaceae | *Stomatium* cf. *suaveolens* Schwantes | 23 |
| Alliaceae | *Tulbaghia acutiloba* Harv. | 61 |
| Amaryllidaceae | *Ammocharis coranica* (KerGawl) Herb. | 53 |
| Amaryllidaceae | *Cyrtanthus macowanii* Baker | 83 |
| Apiaceae | *Alepidea delicatula* Weim. | 87 |
| Apiaceae | *Bupleurum mundii* Cham. & Schltdl. | 17 |
| Apiaceae | *Chamarea* sp. (no further ID possible – leaves absent) | 81 |
| Apocynaceae | *Pachycarpus vexillaris* E.Mey. | 79 |
| Asparagaceae | *Asparagus denudatus* (Kunth) Baker | 36 |
| Asphodelaceae | *Bulbine abyssinica* A.Rich. | 35 |
| Asphodelaceae | cf. *Haworthia* sp. | 47 |
| Asphodelaceae | *Trachyandra asperata* var. *macowanii* (Baker) Oberm. | 27 |
| Asteraceae | *Berkheya cardopatifolia* (DC.) Roessler | 13 |
| Asteraceae | *Felicia fascicularis* DC. | 40 |
| Asteraceae | *Helichrysum aureum* (Houtt.) Merr. var. *aureum* | 38 |
| Asteraceae | *Helichrysum miconiifolium* DC. | 77 |
| Asteraceae | *Helichrysum tysonii* Hilliard | 51 |
| Asteraceae | *Dimorphotheca caulescens* Harv. | 86 |
| Asteraceae | *Osteospermum grandidentatum* DC. | 20 |
| Asteraceae | *Othonna carnosa* Less. var. *carnosa* | 10 |
| Asteraceae | *Senecio coronatus* (Thunb.) Harv. | 16 |
| Asteraceae | *Senecio erubescens* var. *crepidifolius* DC. | 28 |
| Asteraceae | *Senecio gramineus* Harv. | 15 |
| Asteraceae | *Senecio othonniflorus* DC. | 26 |
| Asteraceae | *Senecio ruwenzoriensis* S.Moore | 66 |
| Asteraceae | *Senecio tanacetopsis* Hilliard | 75 |
| Asteraceae | *Stoebe plumosum* L. | 8 |
| Asteraceae | *Tolpis capensis* (L.) Sch.Bip. | 32 |
| Boraginaceae | *Lobostemon stachydeus* DC. & A.DC. | 5 |
| Brassicaceae | *Heliophila* sp. (awaiting ID from MO) | 63 |
| Brassicaceae | *Heliophila rigidiuscula* Sond. | 41 |
| Campanulaceae | *Wahlenbergia krebsii* Cham. subsp. *krebsii* | 24 |
| Campanulaceae | *Wahlenbergia nodosa* (H.Buek) Lammers | 82 |
| Colchicaceae | *Androcymbium striatum* A.Rich. | 25 |
| Crassulaceae | *Crassula* cf. *nudicaulis L.* | 9 |
| Crassulaceae | *Crassula exilis* subsp. *cooperi* (Regel) Tölken | 22 |
| Cyperaceae | *Bulbostylis humilis* (Kunth) C.B.Clarke | 43a |
| Cyperaceae | *Cyperus tabularis* Schrad. | 56 |
| Cyperaceae | *Cyperus usitatus* Burch. var. *usitatus* | 11 |
| Cyperaceae | *Ficinia ramosissima* Kunth | 44 |
| Cyperaceae | *Ficinia stolonifera* Boeck. | 62 |
| Cyperaceae | *Isolepis cernua* (Vahl) Roem. & Schult. var. *cernua* | 43ba |
| Cyperaceae | *Isolepis setacea* (L.) R.Br. | 43bb |
| Cyperaceae | *Schoenoxiphium sparteum* (Wahlenb.) C.B.Clarke | 45 |
| Cyperaceae | *Tetraria fourcadei* Turrill & Schönland | 72 |
| Ericaceae | *Erica passerinoides* (Bolus) E.G.H.Oliv. | 65 |
| Euphorbiaceae | *Adenocline pauciflora* Turcz. | 49 |
| Fabaceae | *Argyrolobium tuberosum* Eckl. & Zeyh. | 37 |
| Fabaceae | *Argyrolobium* sp. | 3 |
| Fabaceae | *Aspalathus acicularis* E.Mey. | 67 |
| Fabaceae | *Indigastrum argyraeum* (Eckl. & Zeyh.) Schrire | 73 |
| Fabaceae | *Indigofera cuneifolia* Eckl. & Zeyh. | 74 |
| Fabaceae | *Indigofera sessilifolia* DC. | 31 |
| Fabaceae | *Indigofera* sp. (awaiting ID from K) | 52 |
| Fabaceae | *Indigofera zeyheri* Spreng. ex Eckl. & Zeyh. | 55 |
| Fabaceae | *Lessertia physodes* Eckl. & Zeyh. | 30 |
| Fabaceae | *Otholobium macradenium* (Harv.) C.H.Stirt. | 4 |
| Fabaceae | *Psoralea margaretiflora* C.H. Stirt. & V.R.Clark | 2 |
| Geraniaceae | *Pelargonium grossularioides* (L.) L'Hér. ex Aiton | 64 |
| Geraniaceae | *Pelargonium tragacanthoides* Burch. | 19 |
| Hyacinthaceae | *Albuca* sp. (awaiting ID from M. M-Az.) | 48 |
| Hyacinthaceae | *Albuca tortuosa* Baker | 60 |
| Hyacinthaceae | *Eucomis autumnalis* (Mill.) Chitt. subsp. *autumnalis* | 95 |
| Hyacinthaceae | *Ornithogalum capillare* J.M.Wood & M.S.Evans | 78 |
| Hypoxidaceae | *Empodium elongatum* (Nel) B.L.Burtt | 6 |
| Hypoxidaceae | *Hypoxis obliqua* Jacq*.* | 46 |
| Iridaceae | *Syringodea concolor* (Baker) M.P.de Vos | 1 |
| Iridaceae | *Dierama robustum* N.E.Br. | 33 |
| Iridaceae | *Moraea spathulata* (L.f.) Klatt | 54 |
| Linaceae | *Linum thunbergii* Eckl. & Zeyh. | 42 |
| Orchidaceae | *Disa porrecta* Sw. | 69 |
| Orobanchaceae | *Harveya huttonii* Hiern. | 84 |
| Poaceae | *Digitaria* sp. | 76 |
| Poaceae | *Merxmuellera stricta* (Schrad.) Conert | 14 |
| Poaceae | *Microchloa kunthii* Desv. | 58 |
| Poaceae | *Themeda triandra* Forssk. | 50 |
| Polygalaceae | *Muraltia* cf. *alopecuroides* (L.) DC. | 88 |
| Polygalaceae | *Polygala microlopha* DC. | 12 |
| Proteaceae | *Faurea* sp. nov. | 90 |
| Restionaceae | *Rhodocoma capensis* Steud. | 21 |
| Rosaceae | *Cliffortia montana* Weim. | 71 |
| Rosaceae | *Rubus ludwigii* Eckl. & Zeyh. subsp. *ludwigii* | 29 |
| Rutaceae | *Acmadenia* sp. nov. | 80 |
| Rutaceae | *Agathosma* sp. nov. | 85 |
| Rutaceae | *Agathosma venusta* (Eckl. & Zeyh.) Pillans | 94 |
| Santalaceae | *Thesium imbricatum* Thunb. | 57 |
| Scrophulariaceae | *Bartsia trixago* L. | 70 |
| Scrophulariaceae | *Jamesbrittenia crassicaulis* (Benth.) Hilliard | 18 |
| Scrophulariaceae | *Nemesia floribunda* Lehm. | 34 |
| Scrophulariaceae | *Selago dolosa* Hilliard | 7 |
| Sterculiaceae | *Hermannia coccocarpa* (Eckl. & Zeyh.) Kuntze | 39 |
| **Photographic Records (voucher specimens not collected)**  <https://picasaweb.google.com/116442385816096754015/BiodiversityScientistsExploreThePoorlyKnown KamdeboobergeInTheGreatKaroo> | | |
| Amaryllidaceae | *Strumaria gemmata* Ker Gawl. |  |
| Asphodelaceae | *Aloe broomii* Schönland |  |
| Asteraceae | *Chrysocoma ciliata* L. |  |
| Asteraceae | *Helichrysum montanum* DC. |  |
| Asteraceae | *Senecio hastatus* L. |  |
| Crassulaceae | *Cotyledon orbiculata* L. |  |
| Fabaceae | *Acacia karroo* Heyn |  |
| Fabaceae | *Trifolium burchellianum* Ser. subsp. *burchellianum* |  |
| Euphorbiaceae | *Euphorbia caterviflora* N.E.Br. |  |
| Oxalidaceae | *Oxalis depressa* Eckl. & Zeyh. |  |
| Scrophulariaceae | *Harveya huttonii* Hiern |  |
| Scrophulariaceae | *Sutera halimifolia* (Benth.) Kuntze |  |
