# Supplementary material for: A rapid multi-disciplinary biodiversity assessment of the Kamdebooberge (Sneeuberg, Eastern Cape, South Africa): implications for conservation
Source: Springerplus. 2012 Dec 6;1(1):56. doi: 10.1186/2193-1801-1-56 (PMC3540356; doi:10.1186/2193-1801-1-56)
Supplement: Supplementary file 2 — Additional file 2: Appendix 2. Tetrapod vertebrates recorded from the Kamdebooberge (22–25 January 2011). (DOC 44 KB) [file 40064_2012_45_MOESM2_ESM.doc]

Appendix 2: Tetrapod vertebrates recorded from the Kamdebooberge (22–25 January 2011).

| **Class** | **Family** | **Species** | **Common Name(s)** | **Area to which the**  **species is endemic*** |
| --- | --- | --- | --- | --- |
| Amphibia | Pyxicephalidae | *Cacosternum boettgeri* | Boettger's Caco (Dainty Frog) | sA &, iso in nA |
| Amphibia | Pyxicephalidae | *Strongylopus grayii* | Clicking (Gray's) Stream Frog | SA |
| Amphibia | Bufonidae | *Amietophrynus rangeri* | Raucous (Ranger's) Toad | SA |
| Reptilia | Cordylidae | *Cordylus cordylus* | Cape Girdled Lizard | WC & EC |
| Reptilia | Cordylidae | *Pseudocordylus microlepidotus* | Cape Crag Lizard | WC, EC, KZN & Mp |
| Reptilia | Gekkonidae | *Afroedura karroica* | Karoo Flat Gecko | Ext Sn |
| Reptilia | Scincidae | *Trachylepis homalocephala* | Red-sided Skink | sA |
| Reptilia | Varanidae | *Varanus albigularis* | Rock (White-throated) Monitor | Afr |
| Aves | Accipitridae | *Aquila verreauxii* | Verreauxs' Eagle | Afr, iso wA |
| Aves | Accipitridae | *Buteo vulpinus* | Steppe Buzzard | migrant |
| Aves | Accipitridae | *Milvus migrans* | Black (Yellow-billed) Kite | migrant |
| Aves | Cisticolidae | *Cisticola lais* | Wailing Cisticola | Afr |
| Aves | Falconidae | *Falco amurensis* | Amur Falcon (Eastern red-footed Kestrel) | migrant |
| Aves | Fringillidae | *Serinus canicollis* | Cape Canary | sA |
| Aves | Hirundinidae | *Hirundo cucullata* | Greater Striped Swallow | Afr |
| Aves | Hirundinidae | *Hirundo fuligula* | Rock Martin | Afr & wA |
| Aves | Apodidae | *Tachymarptis melba* | Alpine Swift | Afr |
| Aves | Motacillidae | *Macronyx capensis* | Cape (Orange-throated) Longclaw | sA |
| Aves | Muscicapidae | *Cercomela familiaris* | Familiar Chat | Afr |
| Aves | Sturnidae | *Onychognathus morio* | Red-winged Starling | eA & sA |
| Aves | Threskiornithidae | *Bostrychia hagedash* | Hadeda Ibis | Afr |
| Mammalia | Bovidae | *Oreotragus oreotragus* | Klipspringer | Afr |
| Mammalia | Bovidae | *Pelea capreolus* | Grey Rhebok | SA |
| Mammalia | Hystricidae | *Hystrix africaeaustralis* | Cape Porcupine | Afr |
| Mammalia | Procaviidae | *Procavia capensis* | Rock Hyrax (Rock Dassie) | Afr & ME |
| * distribution data from Minter et al. (2004) for Amphibia, Branch (1998) for Reptilia, Hockey et al. (2005) for Aves and Skinner and Chimimba (2005) for Mammalia; iso – isolated populations, Afr – Africa, eA – eastern Africa, EC – Eastern Cape, Ext Sn – an extended Sneeuberg range, KZN – KwaZulu-Natal, ME – Middle East, Mp – Mpumalanga, nA – northern Africa, SA - South Africa, Lesotho & Swaziland, sA - southern Africa, wA – western Asia, WC – Western Cape. | | | | |
