# Supplementary material for: A rapid multi-disciplinary biodiversity assessment of the Kamdebooberge (Sneeuberg, Eastern Cape, South Africa): implications for conservation
Source: Springerplus. 2012 Dec 6;1(1):56. doi: 10.1186/2193-1801-1-56 (PMC3540356; doi:10.1186/2193-1801-1-56)
Supplement: Supplementary file 3 — Additional file 3: Appendix 3. Leafhoppers (Cicadellidae), planthoppers (Dictyopharidae, Nogodinidae, Tropiduchidae) and treehoppers (Membracidae) collected on the Kambebooberge (22–25 January 2011). (DOC 54 KB) [file 40064_2012_45_MOESM3_ESM.doc]

Appendix 3: Leafhoppers (Cicadellidae), planthoppers (Dictyopharidae, Nogodinidae, Tropiduchidae) and treehoppers (Membracidae) collected on the Kambebooberge (22–25 January 2011). Collection method: * = fogging; all other by sweeping.

| **Family** | **Subfamily** | **Taxon** | | **Feeding plant** |
| --- | --- | --- | --- | --- |
| Cicadellidae | Typhlocybinae | | *Accacidia improvisa* Dworakowska | *Acacia karroo** |
| Cicadellidae | Eupelicinae | | *Afralycisca umbrina* (Linnavuori) | Forbs |
| Cicadellidae | Agalliinae | | *Austroagallia* sp.n. | *Chrysocoma ciliata*, *Cliffortia montana* |
| Cicadellidae | Deltocephalinae | | *Balclutha* *auranticula* (Naudé) | Grass |
| Cicadellidae | Ulopinae | | *Cephalelus attenuatus* Davies | *Rhodocoma capensis* |
| Cicadellidae | Typhlocybinae | | *Chlorita cylindrica* (Naudé) | *Chrysocoma ciliata, Erica* sp. |
| Cicadellidae | Typhlocybinae | | *Chlorita exilis* (Naudé) | Restios and shrubs |
| Cicadellidae | Deltocephalinae | | *Circulifer karrooensis* (Cogan) | *Chrysocoma ciliata, Cliffortia montana, Chrysocoma ciliata, Erica* sp., *Euryops annae, Rhodocoma capensis,* |
| Cicadellidae | Deltocephalinae | | *Circulifer struthiola* (Cogan) | *Cliffortia montana*, *Rhodocoma capensis* |
| Cicadellidae | Ulopinae | | *Coloborrhis corticina* Germar | *Acacia karroo* ^*^ |
| Cicadellidae | Drakensbergeninae | | *Drakensbergena gigascutica* Stiller | *Merxmuellera* sp. |
| Cicadellidae | Typhlocybinae | | *Empoasca* sp. | *Acacia karroo* ^*^ |
| Cicadellidae | Typhlocybinae | | *Empoascanara* *ethiopica* | Grass |
| Cicadellidae | Deltocephalinae | | *Exitianus nanus* (Distant) | Grass |
| Cicadellidae | Deltocephalinae | | *Exitianus* *taeniaticeps* (Kirchbaum) | Grass |
| Cicadellidae | Ledrinae | | *Hangklippia* *signata* (Linnavuori) | *Agathosma venusta*, *Cliffortia montana, Passerina montana* |
| Cicadellidae | Typhlocybinae | | *Iseza* sp. | *Ehretia rigida* ^*^ |
| Cicadellidae | Deltocephalinae | | *Jannius mecus* Theron | Grass |
| Cicadellidae | Coelidiinae | | *Modderena* sp.n. near *albicosta* Theron | Forbs and shrubs |
| Cicadellidae | Typhlocybinae | | *Molopopterus* near *damus* Theron | *Euryops annae* |
| Cicadellidae | Typhlocybinae | | *Molopopterus obliquus* Theron | *Otholobium macradenium*, *Passerina montana* |
| Cicadellidae | Deltocephalinae | | *Naudeus bivittatus* (Naudé) | Grass |
| Cicadellidae | Deltocephalinae | | New genus and species #1, Athysanini, | *Chrysocoma ciliata*, *Cliffortia montana,* *Passerina montana* |
| Cicadellidae | Deltocephalinae | | New genus and species #2, Athysanini | *Passerina montana, Euryops annae* |
| Cicadellidae | Deltocephalinae | | New genus and species #3, Athysanini | *Ehretia rigida* ^*^ |
| Cicadellidae | Deltocephalinae | | *Paradorydium* sp. | Grass (*Aristida* sp.) |
| Cicadellidae | Deltocephalinae | | *Pravistylus eductus* (Naudé) | Grass |
| Cicadellidae | Deltocephalinae | | *Recilia* sp.n. | Grass |
| Cicadellidae | Deltocephalinae | | *Teinopterus mikrophallus* Stiller | *Merxmuellera* sp. |
| Cicadellidae | Deltocephalinae | | *Tetartostylus angulatus* Linnavuori | Grass |
| Cicadellidae | Deltocephalinae | | *Tetramelasma litopyx* Stiller | *Merxmuellera* sp. |
| Cicadellidae | Deltocephalinae | | *Tzitzikamaia* sp. | Forbs |
| Cicadellidae | Deltocephalinae | | *Vilargus pumilicans* (Naudé) | Grass |
| Dictyopharidae |  | | *Menenches decuma* Fennah | Forbs |
| Dictyopharidae |  | | *Menenches morta* Fennah | Forbs |
| Membracidae |  | | Nymph | Forbs |
| Nogodinidae |  | | *Telmessodes proconsul* Fennah | Forbs |
| Tropiduchidae |  | | *Turneriola* sp. | Forbs |
